# Attitudes Towards World War II Collaboration in Belgium: Effects on Political Positioning Towards the Amnesty Issue in the Two Main Linguistic Communities

**DOI:** 10.5334/pb.346

**Published:** 2017-11-21

**Authors:** Laura De Guissmé, Simona Lastrego, Patricia Mélotte, Laurent Licata

**Affiliations:** 1 Université libre de Bruxelles, BE; 2 FNRS-FRS (FRESH), BE

**Keywords:** Amnesty for collaboration, Intergroup relations, Social identity World war II, Collective memories, Belgium

## Abstract

It is a known fact that some Belgians collaborated with the Nazi occupier during WWII. However, according to a popular myth, collaboration was widespread in Flanders, whereas Walloons bravely resisted. Of course, historical reality is much more nuanced, but this oversimplification has largely resurfaced in political debates surrounding the Belgian linguistic conflict. Demands for amnesty for former collaborators addressed by Flemish nationalist parties are a case in point. We conducted two studies in order to investigate Belgians’ attitudes towards this political issue in the two linguistic communities. In 2012, a first survey (N = 521; 315 French-speakers (FS) and 206 Dutch-speakers (DS)) showed that WWII collaboration was morally condemned, and attitudes towards amnesty were predominantly negative, in both groups. However, DS tended to support amnesty more than FS. This effect of Linguistic Group on Support for Amnesty was mediated by Judgments of Morality of collaboration, and this mediation was moderated by Linguistic identification. In 2015, a second survey (N = 774; 476 FS and 298 DS) confirmed these results. Moreover, judgments about the Unfairness of the repression of collaboration also mediated the effect of Linguistic Group on Support for Amnesty. These results suggest that differences in political position-taking regarding the granting of amnesty between DS and FS are, at least partly, due to different attitudes towards collaboration and to the membership to a linguistic community.

## Introduction

Ordinary people do not think about history as historians (Klein, 2013). Indeed, they make sense out of it by making up new narratives that are not necessarily factually trustworthy and use them “to appraise the present and act on it” (p. 26). Belgians are no exception and for example, as suggested by Gotovitch and Kesteloot (2002), they have a binary vision of their involvement during World War II, where Flemings are perceived as mainly engaged in collaboration whereas Walloons bravely resisted (Benvindo & Peeters, 2009). Of course, historical reality is much more nuanced.

Since the end of WWII, diverging historical representations have heavily weighted on relations between the North and the South of the country. Indeed, “since the second half of the fifties, Belgium cultivates a double memory of the war and its inheritance, and in particular the inheritance of collaboration anchors as a parasite in the Belgian national conflict” (Van Doorslaer, 2006, pp. 482–483). This, in turn, fuels the existing tensions between the two linguistic communities, which has consequently resulted in a difficulty to unify as citizens of a same country (Gotovitch & Kesteloot, 2002). Subsequently, Flanders and Wallonia have grown apart, resulting, among other things, in distinct political priorities. For example, some Flemish political parties have, for decades, requested that the amnesty for former collaborators be examined and discussed at the Belgian parliament. Amnesty pertains to the granting of a general pardon for past (political) offenses, to a group of persons as a whole. According to Verdussen and Degrave (2007, p. 346), the amnesty is a “collective measure that removes the infringement character of some criminally reprehensible facts […] It is a matter of bringing the offense into the oblivion of the social group”. Notably, all French-speaking parties have opposed this idea of amnesty for former collaborators, and in 2011, the Walloon parliament unanimously voted a resolution to support “the duty to remember” and to oppose “any general law of amnesty”.

Some political actors do not hesitate to exploit historical facts or, more precisely, the representation of these facts to gain voters’ appreciation. This allegation can be illustrated by the fact that during the federal elections, the French-speaking side often emphasised the association between Flanders and Nazism. For example, Olivier Maingain (leader of the FDF, a French-speaking party) described the refusal of the Flemish regional government to nominate French-speaking Mayors of Flemish councils as “*a reminder of the Occupation*”. On the Dutch-speaking side, Bart De Wever (leader of the N-VA, a Flemish party) denounced “the Walloon myth” according to which resistance was mainly the fact of Walloons, while Flemings were largely engaged in collaboration: “It’s better anyway to shed light on the past of a society without masking the reality than judging from a misplaced moral superiority, and based on collective ignorance” (*Le Soir*, 2010). However, in May 2015, in front of the Jewish community, he stated that “Collaboration was a terrible mistake at all levels” (*Le Soir*, 2015).

As suggested by Liu and Hilton (2005), the past heavily weights on the present and can determine a group’s identity. Thus, not sharing the same past, or more precisely the perception of it, undermines the sense of unity among Belgian citizens (Hirst & Fineberg, 2011). Furthermore, according to Jacobs and Khanna (2012), the disagreements between the two main linguistic communities currently threaten the very existence of Belgium. Therefore, the time has come to look into the attitudes towards WWII collaboration that predominate on both sides of the linguistic border. This research is especially important because the myth of the Walloon resistant and of the Flemish collaborationist does not hold in front of historical facts and does not help to reduce the existing tensions between the linguist communities (De Wever, 2006; Plisnier, 2008). To the best of our knowledge, Belgian lay people’s representations of WWII collaboration have never been empirically investigated. Thus, this paper will examine to what extent, and how, French and Dutch-speaking representations of WWII collaboration influence current political positioning about the amnesty of collaborators.

### Dealing with past misdeeds as a group

Roccas, Klar, and Liviatan (2004, p. 131) have argued that “confronting information that indicates one’s ingroup has committed acts that are incompatible with one’s moral standards is an unpleasant psychological experience for most if not all group members” and can result in important consequences on self-esteem and on emotional wellbeing (Branscombe & Doosje, 2004). Thus, it is not surprising that French-speakers have cleared their consciences by “erasing” Wallonia’s collaborative past and that Dutch-speakers have been trying to obtain amnesty (Gotovitch & Kesteloot, 2002). Furthermore, as suggested by Jetten and Wohl (2012), the past also influences our future interactions. Thus, if the two linguistic communities do not share the same attitudes towards their past, this can result in current tensions.

The past also contributes to the valorisation of the group’s identity by defining the group’s value through intergroup comparisons (Tajfel & Turner, 1986); again, this could account for the fact that collaboration has been assigned to one community allowing the other one to maintain a positive image by contrast. Indeed, “past successes and failures of the group but also its moral and immoral actions contribute to define its relative value” (Klein, Licata, Van der Linden, Mercy, & Luminet, 2012, p. 18). The moral dimension is considered as the most crucial for achieving group valorisation, compared to other characteristics (Leach, Ellemers, &Barreto, 2007). Accordingly, past events can be reinterpreted in order to preserve or increase perceived in-group morality: historical events that shed a positive light on the ingroup’s morality are remembered, whereas shameful events are forgotten (Sahdra & Ross, 2007). Finally, the past can be used for mobilizing group members for a collective project (Klein & Licata, 2003), as for example to request or to banish amnesty.

As a consequence, group members’ attitudes towards their in-group’s past can have a significant impact on their responses to new crises or challenges, and on their current intergroup relations. Considering the polemical nature of WWII collaboration outlined above, Belgium is a particularly fertile ground for studying the influence of attitudes about the past on current political debates.

### The Belgian case: Some historical facts

Tensions between the linguistic communities have aroused fairly early in Belgium. Indeed, the Belgian elites were mainly French-speaking throughout the country and Dutch became an official language only in 1898. Since the creation of Belgium as a state in 1830, Flemings have been striving for more cultural and linguistic recognition. This struggle eventually evolved into a sub-nationalist movement that requested more political and economic autonomy, or even secession. In contrast, although some parts of the Walloon movement also requested more regional autonomy during the first half of the 20^th^ century, most French-speakers have supported the unity of the country. This incompatibility of views regarding the future of the country has led to several political crises. The most acute one led to a period of 541 days without a government in 2010–2011.

These differences in the historical experiences of the two linguistic groups help explain why the French-speaking identity tends to be seen as compatible with the Belgian national identity, whereas Dutch-speaking identity tends to be seen as incompatible with it. Research has shown that French-speakers tend to identify more with Belgium than with their linguistic group, whereas the opposite trend has often been reported among Dutch-speakers (e.g. De Winter, Frognier, & Billiet, 1998; Doutrelepont, Billiet, & Vandekeere, 2001; Rimé et al., 2015).

### Collaboration during World War II

During WWII, the Vlaams Nationaal Verbond (VNV, a Flemish nationalist party, 15% in 1939 elections) supported the occupier and collaborated in the hope to obtain an independent Flemish state (Dujardin & Van den Wijngaert, 2010). In Flanders, military and political collaboration were less contested than in Wallonia, where resistance was more developed (Kesteloot, 2013). However, there were also collaboration movements in Wallonia: Léon Degrelle, the leader of Rex (a catholic far-right party, 6% in 1939 elections) also collaborated with the Nazis. However, the collaborators’ profiles were globally different in the two linguistic communities. In Flanders, some prominent collaborators were intellectuals and politicians defending the Flemish movement, whereas, in Wallonia, most collaborators were seen as criminals. Others, associated with the Nazi ideology, were perceived as traitors (Beyen, 2002).

As a consequence, during and after the war, collaborators were generally hated in Wallonia (Gotovitch & Kesteloot, 2002). The situation was more ambiguous in Flanders, where collaborators were perceived as victims of the post-war repression by a significant part of the public opinion, and where some collaborators were reintegrated in political positions (Kesteloot, 2013). In addition, according to Heenen-Wolff, Bazan, and Verougstraete (2012), the brutal repression of presumed collaborators at the liberation greatly damaged (or even, traumatized) the Flemish identity. Some presumed collaborators fell prey to popular vindictiveness: they were publicly brutalized, some women were shaved, etc. The view that the Belgian State did not play its role because it failed to protect presumed collaborators and their families also contributes to shape Flemish attitudes towards this period. However, contrary to a widespread belief in Flanders, historians have shown that the legal repression of collaborators at the liberation was not more severe in Flanders than in Wallonia (Aerts, 2011). Finally, in contrast with some political discourses in favour of amnesty, some measures have already been taken to the benefit of collaborators (releases on parole and measures of royal pardon). However, it is worth noting that the history of collaboration during WWII was discussed in the Flemish public sphere. Far from being consensual, there exist acute dissensions about this past among Flemings.

In contrast, in Wallonia, collaborators were excluded from the political realm, and the struggle against fascism was considered as the basis of the post-war Walloon identity. As a consequence, the Walloon collaboration was generally forgotten, or even became a real taboo (Balace, 2002). In collective memories, collaboration was associated with Flemings while resistance was associated with Walloons.

These differences in how WWII history was processed in the two linguistic groups help explain the divergence in political position-taking towards the amnesty issue. Indeed, requesting amnesty for collaboration may be perceived as a legitimate claim in some segments of Flemish public opinion, and is in line with an effort for building a sense of positive social identity at the regional/linguistic level. In fact, as previously described, a negative group identity is highly detrimental for individuals’ wellbeing. In contrast, these claims may seem utterly illegitimate on the French-speaking side, where they come into conflict with the Walloon identity, partly based on the resistance ideology. As a consequence, these diverging WWII memories impede the construction of a common Belgian memory, and therefore also that of a common national identity.

The dynamics described above are based on historical inquiries and on analyses of political and media discourses (e.g. Gotovitch & Kesteloot, 2002). So far, little, if anything, is known about the way lay people perceive the history of Belgian collaboration and how members of the two linguistic communities position themselves regarding the amnesty debate.

In the present paper, we investigate how Belgians represent WWII collaboration in Belgium by taking into account their linguistic affiliation (French or Dutch speakers). More generally, we also aimed to test the theoretical hypothesis that attitudes towards the past shape the way people take positions in current political debates, such as amnesty requests. Study 1 tests a first theoretical model (see below) that links linguistic group belonging to political standpoints towards amnesty, judgments of immorality of collaboration, and identification with the linguistic group and with Belgium. Study 2 supports this model by replicating the results of Study 1 and investigates two new variables: judgment of unfairness of the repression of collaboration and judgment of the role of the State in containing popular vindictiveness.

## Study 1

Since understanding people’s attitudes towards the past can improve our understanding of the issues of current society (Liu & Hilton, 2005), we designed a first study to investigate how attitudes towards collaboration with the German occupiers during WWII in Belgium relate to position-taking in the current political debate about the amnesty of former collaborators in the two linguistic groups. Because the collaborationist past of Belgians has been distorted in a way that tends to attribute collaboration only to the Flemish part of the country, we hypothesized that French-speaking respondents would judge collaboration as more immoral (H1), and would express less support for amnesty than Dutch-speaking respondents, as the latter need to restore their group’s identity (H2). In addition, we hypothesized that the Judgment of Immorality of collaboration would be negatively correlated with Support for amnesty in both linguistic communities because those who despise collaboration are generally against amnesty (H3). We then predicted that differences in moral judgments on collaboration would mediate the intergroup difference in support for amnesty (H4). Furthermore, and in line with the findings of previous research (Klein et al., 2012; Leach et al., 2007), we expected that participants’ identification with their linguistic community would moderate this effect: because people need to maintain a positive image of their group, the above hypothesis should hold only for people who identify with their linguistic group (we thus expected no relations whatsoever for those who do not identify with their linguistic group) (H5).

Finally, and as stated above, attitudes towards WWII are strongly connected to political discourses in the North and the South of the country. In Flanders, right-wing parties have been requesting amnesty since decades. In the French-speaking side of the country, only far-right parties have expressed sympathy for former collaborators. We thus hypothesized that right-wing political orientation would be positively correlated with Support for amnesty (H6) in the two groups.

### Method

**Participants.** Five hundred and fifty Belgian participants completed the questionnaire in French or in Dutch. Five hundred and twelve participants completed the online version and thirty-eight participants completed the paper version (the questionnaire was printed in order to allow some elderly participants to participate in the study). The only criterion for participating was to hold the Belgian nationality. Four participants were discarded from analyses because they did not fulfil this condition and twenty-five others because they completed the questionnaire in French or in Dutch although they identified themselves as members of the other linguistic group. The final sample thus comprised 521 participants: 315 French-speakers (FS, 170 men and 145 women) and 206 Dutch-speakers (DS, 171 men and 35 women). Participants were aged from 17 to 94 years old: the most represented age group among FS was the “17 to 24 years old” (23,8%) whereas the “45–54 years old” (26,7%) was the most represented among DS. In order to control for these differences of age and gender, we included these variables in the following analyses. Almost sixty percent of participants were left-wing voters (people who indicated that they are left, far left or centre left voters; 59.6% of FS and 59.7% of DS). This is in line with actual election results in Wallonia, which is predominantly left-wing, but it is at odds with those in Flanders, where the majority usually votes for right-wing parties. For this reason, political orientation will be controlled for in subsequent analyses.

**Procedure.** In 2012, participants were recruited through postings on Facebook groups dedicated to World War II and to Belgian political parties. The invitation to participate in a study about “Representations and collective memories of resistance and collaboration in Belgium during WWII” comprised a link to an online questionnaire. The CEGES/SOMA (Centre for historical research and documentation on war and contemporary society) agreed to place the invitation in both languages on its website. The “Centrale Générale FGTB – Algemene Centrale ABVV”, a labour trade union, also agreed to disseminate the invitation to participate. Finally, we also used the snowballing method to spread the questionnaire and also gave the possibility to complete a paper version. All participants participated on a voluntary basis.

**Measures.** Participants answered the questions in the following order. All items were assessed on 7-point scales from 1 (Entirely disagree) to 7 (Entirely agree).

***National and linguistic identifications.*** Three items, adapted from Brown, Condor, Mathews, Wade, and Williams (1986), measured the extent to which respondents identified with Belgium: “I am proud to say that I am Belgian”, “Most of the time, I like to think of myself as a Belgian”, “I feel attached to Belgium”: α_FS_ = .91 and α_DS_ = .93. Next, three items using similar scales measured the extent to which respondents identified with their linguistic community: “I am proud to say that I am FS/DS”, “Most of the time, I like to think of myself as a FS/DS”, “I feel attached to Wallonia/Flanders”: α_FS_ = .81 and α_DS_ = .94.

***Immorality of collaboration.*** One single item assessed participants’ judgment of the degree of immorality of WWII collaboration: “To what extent do you judge WWII collaboration as immoral?”

***Support for amnesty.*** Twelve items assessed participants’ attitudes about amnesty for collaborators (e.g. “According to me, we should grant amnesty to collaborators”, “Granting amnesty for collaborators is unacceptable” (reversed), “Granting amnesty would be an insult to the memory of WWII victims” (reversed), “If a referendum on amnesty was organized, I would vote in favour [of amnesty]”): α_FS_ = .86 and α_DS_ = .95.

***Political orientation.*** Participants provided information about their political orientation from 1 (Far left) to 7 (Far right).

***Demographic information.*** Participants provided information about their age, gender, mother tongue, education and socio-economic situation.

### Results

**Preliminary results.** Means, standard deviations, and correlations are presented in Table 1. We conducted independent t-test to compare means between FS and DS. As expected (H1), FS judged collaboration as more immoral, and expressed less Support for amnesty (H2) than DS. In addition, in agreement with previous research (e.g. De Winter et al., 1998; Doutrelepont et al., 2001), Identification with Belgium was stronger among FS than DS. However, contrary to these studies, FS also – marginally – tended to identify more with their linguistic group than DS respondents.

**Table 1:** Descriptive Statistics and paired t-tests by Group (Below diagonal: French-speaking respondents and above diagonal: Dutch-speaking participants).

Study 1	1		2		3		4		5		6		7		M_FS_	SD_FS_		M_DS_	SD_DS_	*t*(519)	*p*

**1. Identification to Belgium**	1		–.27	***	.45	***	–.48	***	–.35	***	.09		–.11		5.16		1.57	4.15	1.79	6.62	**<.001**
**2. Linguistic identification**	.44	***	1		–.35	***	.44	***	.51	***	–.20	**	.10		4.61		1.65	4.34	1.83	1.68	.093
**3. Immorality**	.14	*	.19	***	1		–.58	***	–.49	***	<.01		.11		5.60		1.40	4.79	1.85	5.32	**<.001**
**4. Support for amnesty**	–.14	*	–.27	***	–.48	***	1		.48	***	–.03		–.12		2.59		1.16	3.61	1.81	–7.16	**<.001**
**5. Political orientation**	.10		.02		–.14	*	.07		1		–.15	*	<–.01		3.30		1.58	3.28	1.53	.11	.92
**6. Gender**	.07		.02		–0.08		.19	***	–.09		1		–.19	**	46% ♀			17% ♀			
**7. Age**	.12	*	.20	***	.30	***	–.33	***	–.04		–.31	***	1		4.05		1.71	5	1.59	–6.53	**<.001**

*Note*: ***p ≤ .001, **p ≤ .01, *p ≤ .05.

Furthermore, as expected (H3), Immorality of collaboration was negatively correlated with Support for amnesty among FS and DS, while a more right-wing political orientation (H6) positively correlated with Support for amnesty among DS, but not among FS.

It is worth noting, however, that the means for Immorality of collaboration were significantly higher than the midpoint of the scale in the two groups (*t_FS_*(314) = 20.21, *p* < .001; *t_DS_*(205) = 6.13, *p* < .001). And the means for Support for amnesty were significantly lower than the midpoint of the scale in both groups (*t_FS_*(314) = –21.54, *p* < .001; *t_DS_*(205) = –3.07, *p* < .01). Thus, on average, both FS and DS condemned collaboration and opposed amnesty, but FS tended to do so more strongly than DS.

In addition, the two levels of identification correlated positively among FS, but negatively among DS (see Table 1), indicating that linguistic identification tends to be viewed as compatible with national identity among FS, while it appears to be incompatible among DS. Political orientation strongly correlated with Identification in the Dutch-speaking group but not in French-speaking group, suggesting that identification patterns differed as a function of this variable only for the DS group. Correlations among DS indicated that the more they positioned themselves on the right side of the political spectrum, the more they identified as Dutch-speakers, and the less they identified as Belgians. This reveals an important distinction in DS identification patterns in relation with their political orientation, whereas identification patterns are much more consensual among FS.

Finally, descriptive results indicated that age group, gender, and political orientation were associated with Support for amnesty for both DS and FS. Therefore, we controlled these variables in the next analysis.

**Figure 1:**
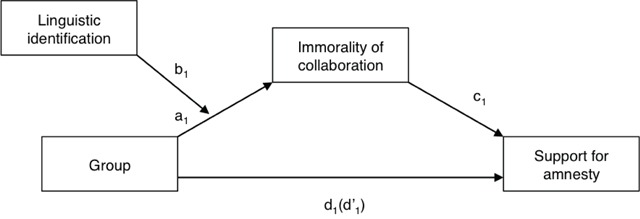
Moderated mediation model explaining the effect of Group on Support for amnesty (Study 1).

**Moderated mediation.** Using the PROCESS macro developed by Hayes (2012, Model 7, see Figure 1), we finally tested H4; the effect of the Linguistic Group on Support for Amnesty mediated by Immorality of collaboration, and H5; the moderation of this mediation by Linguistic identification, through a moderated mediation model. In this model, Group (DS vs. FS) was the independent variable, Linguistic identification was the moderator, Immorality of collaboration was the mediator and Support for amnesty was the dependent variable. Political orientation, Age and Gender were also entered as covariates in order to control for their effects. Results, presented in Table 2, show that Group had a significant effect on Immorality of collaboration, which in turn had a significant negative effect on Support for amnesty. The indirect effect proved to be significant, *ab* = .22, *SE* = .04, *95% CI* [.15, .30]. Indeed, the effect of Group on Support for amnesty significantly decreased when the mediator (Immorality of collaboration) was entered in the analysis, although it remained significant. Thus, a partial mediation was observed. Moreover, Linguistic identification moderated the effect of Group on Support for amnesty. Indeed, the stronger Linguistic identification was, the more Group predicted Support for amnesty. This means that the difference between FS and DS in Support for amnesty was greater when participants were highly identified. Finally, analyses of conditional indirect effects indicated that when Group predicted Support for amnesty, Immorality of collaboration mediated this effect, but only among middle and highly identified participants.

**Table 2:** Results for the moderated mediation model explaining the effect of Group on Support for amnesty (Study 1).

	*b*	*S.E.*	*95% CI*

**Total Effect**			
Group to Support for amnesty (d’_1_)	**.62**	**.07**	**[.49, .75]**
**Direct Effect**			
Group to Support for amnesty (d_1_)	**.40**	**.06**	**[.28, .52]**
**Path from IV to mediator**			
Group to Immorality of collaboration (a_1_)	**.38**	**.19**	**[.02, .75]**
Linguistic identification	–.08	.04	[–.15, .01]
Linguistic identification × Group to Immorality of collaboration (b_1_)	**–.20**	**.04**	**[–.28, –.13]**
**Path from mediator to DV**			
Immorality of collaboration to Support for amnesty (c_1_)	**–.43**	**.04**	**[–.50, –.36]**
**Conditional Indirect Effects**			
**Group to Support for amnesty**			
Mediation through Immorality of collaboration – Low Identification	.08	.04	[–.01, .15]
Mediation through Immorality of collaboration – Middle Identification	**.23**	**.04**	**[.16, .31]**
Mediation through Immorality of collaboration – High Identification	**.38**	**.06**	**[.27, .52]**


### Discussion

The aim of this first study was to investigate how attitudes towards collaboration during WWII in Belgium related, among Belgians, with position-taking in the current political debate about the amnesty of former collaborators in the two linguistic groups. This study shows that, on average, collaboration was morally condemned, and attitudes towards amnesty were predominantly negative in both linguistic groups. However, as hypothesised, Dutch-speakers tended to condemn collaboration less strongly, and were relatively more favourably disposed towards amnesty than French-speakers. We suggested that this should be expected as Dutch-speakers have been attributed a greater involvement in the collaboration than French-speakers. Thus, it is not surprising that they are more favourable towards a political act that could restore their image. Interestingly, we also found that the older French- and Dutch- speakers were, the more they morally condemned collaboration and the less they were in favour of amnesty. We could imagine that younger generations have less suffered from this past than elderly generations. Licata and Klein (2010) have indeed shown that perceptions of the past are different according to the age gap between citizens of a same country. Moreover, as expected, political orientation had an impact on immorality of collaboration and support for amnesty, especially among Dutch-speakers: the more they positioned themselves on the right side of the political spectrum, the less they judged collaboration as immoral, and the more they supported amnesty. Similarly, the more French-speaking respondents positioned themselves on the right side of the political spectrum, the less they judged collaboration as immoral. But rejection of amnesty was consensual across the whole French-speaking political spectrum, with the only exception of a small number of far-right voters. This last result strengthens past findings suggesting that French-speakers do not feel that collaboration concerned their group and thus are not favourable to amnesty requests (Gotovitch & Kesteloot, 2002).

Interestingly, and in accordance with Roccas et al.’s (2004) idea that ingroup involvement in immoral behaviours puts group members in a dangerous position, we found that national and linguistic identifications had opposite impacts on judgments of immorality of collaboration and support for amnesty among Dutch-speaking respondents. The more they identified as Belgians, the more they morally condemned collaboration and the less they supported amnesty. In contrast, the more they identified as Dutch-speakers, the less they condemned collaboration and the more they supported amnesty. Depending on the group they identify with, Dutch-speakers tune their moral judgment and thus preserve the morality of their group. However, this was not the case among French-speaking respondents: both levels of identification had similar negative effects on these two variables, probably because whichever group they relate to, collaboration is not a threat to their identity. Moreover, among French-speaking respondents, we found that the more they felt Walloon (or Brusseler), the more they felt Belgians. The French-speaking linguistic identity tends to be seen as compatible with the Belgian national identity. However, the opposite trend was observed among Dutch-speakers: the Flemish identity tends to be seen as incompatible with the Belgian national identity. Moreover, political orientation had a polarizing effect on Dutch-speaking respondents: the more they positioned themselves on the right side of the political spectrum, the more they identified with the Flemish community, and the less they felt Belgian.

Furthermore, the mediation analyses have shown that Flemings who mildly or highly identified with their linguistic group tended to judge collaboration as less immoral than French-speakers, and therefore expressed more support for amnesty. On the contrary, mildly and highly identified French-speakers tended to judge collaboration as more immoral than did Dutch-speakers and, therefore, to oppose amnesty. There was no difference between weakly identified members of the two groups.

Although these first results allow us to further understand the divergent attitudes of Dutch and French-speakers towards amnesty, we must acknowledge that this study suffered from some limitations. First, our sample was mainly composed of leftist participants, whereas the majority in Flanders tends to vote for right-wing parties. Our results showed that political orientation had an important impact on judgments of collaboration. Although we systematically controlled for the effect of this variable in Study 1’s analyses, we aimed to replicate our results in a sample more in line with the political orientations of the two linguistic groups at stake.

Another caveat is that Immorality of collaboration was measured through a single item, which might be less reliable than multiple item scales. We therefore decided to include more items in a second study.

Finally, as stated above, according to historians, a portion of the Flemish opinion tends to view the repression of collaboration as an injustice committed by the State. Perception of the relative fairness of the repression could therefore influence political position-taking towards amnesty of collaborators, over and above judgments on the immorality of collaboration itself. Another factor stressed by historians (e.g. Van Loon, 2008) is the perception that the Belgian State failed to protect presumed collaborators from popular vindictiveness at the liberation. Indeed, some (presumed) collaborators were brutalized by local populations, and women accused of having had intimate relationships with German soldiers were publicly humiliated. This stands in stark contrast with the application of justice in a state subjected to the rule of law, and might have generated a feeling of injustice, especially in Flanders (Heenen-Wolff et al., 2012). The perception that the State failed to play its role at the liberation, and its failure to recognize the harm done to presumed collaborators, should lead to more Support for amnesty of collaborators. We therefore introduced measures of perception of the Unfairness of repression of collaboration in a second study, as well as measures of the perception of the inadequacy of the Role of the Belgian State after the war.

## Study 2

In order to address the limitations of Study 1, we conducted a second cross-sectional study. The first aim of Study 2 was to replicate the results (including the moderated mediation model) found in Study 1 in order to test the robustness of these findings. Hence we expected to replicate that attitudes towards collaboration with the German occupiers during WWII in Belgium relate to position-taking in the current political debate about the amnesty of former collaborators in the two linguistic groups. The second aim was to extend the findings by including two new variables: perception of the Unfairness of the repression of collaboration, and the Role of the State in failing to contain popular vindictiveness against collaborators, because we expect that these perceptions could influence how amnesty is perceived. In particular, we expected that Unfairness of repression would be negatively correlated with Immorality of collaboration (H7a) and positively correlated with Support for amnesty (H7b). The same trends should be observed for Role of the state (H8a and H8b).

### Method

**Participants.** Eight hundred and seven participants completed the questionnaire in French or in Dutch. The only condition for participating was to hold the Belgian nationality. Twelve participants were discarded from analyses because they did not meet this criterion and twenty-one others because they completed the questionnaire in French or in Dutch whilst identifying themselves as members of the other linguistic group. The final sample thus comprised 774 participants: 476 FS (336 men and 140 women, *M_age_* = 45.88, *SD* = 16.86) and 298 DS (253 men and 45 women, *M_age_* = 53.74, *SD* = 15.28). Finally, 53.6% of French-speaking and 51.7% of Dutch-speaking respondents were left-wing voters (thus, 6% and 8% less than in Study 1). In the French-speaking sample, right-wing voters are over-represented in comparison with actual election results. As in Study 1, left-wing voters are again over-represented among the Dutch-speaking sample, although less so than in Study 1.

**Procedure.** In 2015, participants were recruited through the same means as in Study 1. In addition, the French-speaking weekly newspaper “Le Vif/L’express” also agreed to place the invitation on its Facebook page. All respondents participated on a voluntary basis.

**Measures.** Participants answered the questions in the following order. All items were assessed on 7-point scales from 1 (Entirely disagree) to 7 (Entirely agree).

***National and linguistic identifications.*** We used the same measures as in Study 1 to measure national identification (α_FS_ = .92; α_DS_ = .95) and identification with the linguistic group (α_FS_ = .82; α_DS_ = .95).

***Immorality of collaboration.*** Four items assessed participants’ judgment of the immorality of collaboration during WWII (e.g. “In some circumstances, collaboration with Germans was moral” (reversed), “Collaboration during WWII is morally condemnable”): α_FS_ = .60 and α_DS_ = .72.

***Unfairness of the Repression of collaboration.*** Two items assessed participants’ belief about the unfairness of the repression of collaboration after the war in the two linguistic groups (“The repression of French-speaking/Dutch-speaking collaboration was unfair”). The two items were highly correlated in the two sub-samples (*r*_FS_ = .91, *p* < .001 and *r*_DS_ = .91, *p* < .001). We thus created a single scale tapping perception of the unfairness of collaboration across groups.

***Role of the State in containing popular vindictiveness.*** Two items assessed participants’ belief about the fact that the State did not play its role by having been unable to contain the popular vindictiveness after the Second World War, and by having failed to recognize the harm done to its targets: “After the Liberation, the State did not play its role: it did not sufficiently protect people from the popular vindictiveness” and “After the war, the State did not sufficiently recognize the wrongs undergone by people targeted by the popular vindictiveness”: *r*_FS_ = .66, *p* < .001 and *r*_DS_ = .72, *p* < .001.

***Support for amnesty.*** We used the same measure as in Study 1: α_FS_ = .91 and α_DS_ = .95.

***Political orientation.*** Participants provided information about their political orientation from 1 (Far left) to 7 (Far right).

***Demographic information.*** Participants provided information about their age, gender, mother tongue, education and socio-economic situation.

### Results

**Preliminary results.** Means, standard deviations, and correlations are presented in Table 3. As in Study 1, FS judged collaboration as more immoral than DS (H1) and DS expressed more support for amnesty than FS (H2). Moreover, DS judged repression as more unfair than FS. However, there was no significant difference in judgment about the Role of the State just after the war between the two groups. In addition, in agreement with Study 1, Identification with Belgium was stronger among FS than DS, and FS also marginally tended to identify more with their linguistic group than DS respondents.

**Table 3:** Descriptive Statistics and paired t-tests by Group (Below diagonal: French-speaking respondents and above diagonal: Dutch-speaking participants).

Study 2	1		2		3		4		5		6		7		8		9		M_FS_	SD_FS_	M_DS_	SD_DS_	*t*(772)	*p*

**1. Identification to Belgium**	1		–.12	*	.30	***	–.41	***	–.30	***	–.28	***	–.25	***	.02		.10		5.39	1.53	4.96	1.76	3.52	**<.001**
**2. Linguistic identification**	.31	***	1		–.27	***	.37	***	.25	***	.32	***	.55	***	–.18	**	.07		4.66	1.55	4.44	1.88	1.71	.09
**3. Immorality**	.06		.12	*	1		–.61	***	–.43	***	–.31	***	–.35	***	–.11		.21	***	5.47	1.22	5.17	1.36	3.13	**.002**
**4. Support to amnesty**	–.04		–.18	***	–.48	***	1		.56	***	.54	***	.33	***	.05		–.19	***	2.73	1.35	3.67	1.74	–7.94	**<.001**
**5. Unfairness of repression**	.04		–.10	*	–.34	***	.47	***	1		.42	***	.29	***	–.01		–.17	**	2.24	1.45	2.77	1.60	–4.73	**<.001**
**6. Role of the State**	.06		.08		–.13	**	.16	***	.11	*	1		.20	***	.02		.01		4.93	1.40	4.90	1.63	.28	.78
**7. Political orientation**	.12	**	–.10	*	–.29	***	.24	***	.19	***	.15	***	1		–.17	**	–.02		3.55	1.56	3.57	1.43	–.19	.85
**8. Gender**	.05		.06		.09		–.04		–.02		.06		–.19	***	1		–.18	**	29% ♀		15% ♀			
**9. Age**	–.02		.31	***	.08		–.23	***	–.17	***	.05		–.01		–.10	*	1		45.9	16.9	53.7	15.3	–6.54	**<.001**

*Note*: ***p ≤ .001, **p ≤ .01, *p ≤ .05.

Furthermore, Immorality of collaboration negatively correlated with Support for amnesty (H3), and Political orientation positively correlated with Support for amnesty, in both groups (H6).

As expected (H7a and H8a), Unfairness of repression and Role of the State were negatively associated with Immorality of collaboration, and positively associated with Support for amnesty (H7b and H8b) in both groups, although these correlations were stronger among DS. Indeed, Fisher’s Z-tests confirmed that all these correlations were stronger among DS (respectively: *z* = 1.43, *p* = .08; *z* = 2.56, *p* = .005; *z* = 1.66, *p* = .049; *z* = 5.97, *p* < .001).

Once again, it is worth noting that the means for Immorality of collaboration were significantly higher than the midpoint of the scale (for FS, *t*(499) = 26.93, *p* < .001; for DS, *t*(306) = 15.24, *p* < .001), and that the means for Support for amnesty were also significantly lower than the midpoint of the scale, in both groups (for FS, *t*(499) = –21.14, *p* < .001; for DS, *t*(306) = –3.54, *p* < .001).

In addition, and in line with results of Study 1, the two levels of identification correlated positively among FS, but negatively among DS (see Table 3), indicating that linguistic identification tends to be viewed as compatible with national identity among FS, whereas it appears to be incompatible among DS. However, the Political orientation measure strongly and positively correlated with Linguistic Identification in the Dutch-speaking group but it correlated less strongly, and negatively, in the French-speaking group (Fisher’s Z-test: *z* = 6.98; *p* < .001), suggesting that identification patterns differed as a function of political orientation mainly for the DS group. In particular, correlations among DS participants indicated that the more they positioned themselves on the right side of the political spectrum, the more they identified as Dutch-speakers, and the less they identified as Belgians. Correlations among FS indicated that the more they positioned themselves on the right side of the political spectrum, the more they identified as Belgians, and the less they identified as French-speakers.

Finally, age and political orientation were associated with Support for amnesty for both DS and FS. Therefore, we controlled for the effects of these variables in the next analysis.

**Figure 2:**
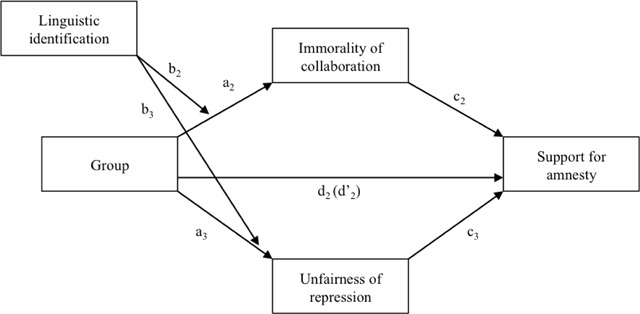
Moderated mediation model explaining the effect of Group on Support for amnesty (Study 2).

**Moderated mediation.** Using the PROCESS macro developed by Hayes (2012, Model 7, see Figure 2), we finally tested the same moderated mediation as in Study 1 but with two mediators: Immorality of collaboration and Unfairness of repression. Role of the State was not included in this analysis because the descriptive analyses reported above showed that it did not differ as a function of Group. Political orientation, Age and Gender were also entered as covariates in order to control for their effects. Results (see Table 4) show that Group had significant effects on the two mediators, which in turn had significant effects on Support for amnesty. The total indirect effect proved significant, *ab* = .19, *SE* = .03, *95% CI* [.13, .26]. Indeed, the effect of Group on Support for amnesty significantly decreased when mediators (Immorality of collaboration and Unfairness of repression) were entered, although it remained significant. A partial mediation was thus observed. The individual indirect effects of Immorality of collaboration, *ab* = .08, *SE* = .02, *95% CI* [.04, .13] and of Unfairness of repression, *ab* = .11, *SE* = .02, *95% CI* [.07, .16] on Support for amnesty proved significant. There was no significant difference between these two paths, *b* = –.02, *SE* = .03, *95% CI* [–.08, .03]. Moreover, Linguistic identification moderated the effect of Group on Support for amnesty. Indeed, as in Study 1, the stronger Linguistic identification was, the more Group predicted Support of amnesty. This means that the difference between FS and DS in Support for amnesty was greater when participants were highly identified. Finally, analyses of conditional indirect effects indicated that when Group predicted Support for amnesty, Immorality of collaboration mediated this effect, but the mediating effect of this variable was significant only among middle and highly identified participants. Furthermore, Unfairness of repression mediated the effect of Group at all levels of Linguistic identification, but the mediating effect of this variable increased with the level of the linguistic identification of the participants.

**Table 4:** Results for the moderated mediation model explaining the effect of Group on Support for amnesty (Study 2).

	*b*	*S.E.*	*95%CI*

**Total Effect**			
Group (d’_2_)	**.56**	**.05**	**[.46, .67]**
**Direct Effect**			
Group (d_2_)	**.35**	**.05**	**[.26, .44]**
**Path from IV to mediators**			
Group to Immorality of collaboration (a_2_)	**–.20**	**.05**	**[–.29, –.11]**
Linguistic identification to Immorality of collaboration	–.01	.03	[–.07, .04]
Linguistic identification × Group to Immorality of collaboration (b_2_)	**–.07**	**.03**	**[–.12, –.02]**
Group to Unfairness of repression (a_3_)	**.33**	**.06**	**[.22, .44]**
Linguistic identification to Unfairness of repression	.04	.03	[–.02, .10]
Linguistic identification × Group to Unfairness of repression (b_3_)	**.08**	**.03**	**[.02, .14]**
**Path from mediator to DV**			
Immorality of collaboration to Support for amnesty (c_2_)	**–.44**	**.04**	**[–.51, –.37]**
Unfairness of repression to Support for amnesty (c_3_)	**.34**	**.03**	**[.28, .39]**
**Conditional Indirect Effects**			
**Group to Support for amnesty**			
Mediation through Immorality of collaboration – Low Identification	.03	.03	[–.02, .09]
Mediation through Immorality of collaboration – Middle Identification	**.09**	**.02**	**[.05, .14]**
Mediation through Immorality of collaboration – High Identification	**.14**	**.03**	**[.08, .21]**
**Conditional Indirect Effects**			
**Group to Support for amnesty**			
Mediation through Unfairness of repression – Low Identification	**.07**	**.03**	**[.01, .12]**
Mediation through Unfairness of repression – Middle Identification	**.11**	**.02**	**[.07, .16]**
Mediation through Unfairness of repression – High Identification	**.16**	**.04**	**[.10, .23]**


### Discussion

Study 2 globally replicated Study 1’s results. In addition, two variables about the immediate post-war period and the repression of collaborators that took place in Belgium were added. Results showed that Dutch-speaking participants judged the repression of collaboration as more unfair than French-speaking participants. However, judgments about the inappropriate reaction of the State did not differ between the two linguistic groups. Further, Unfairness of repression positively predicted Support for amnesty in the two groups, and so did Role of the State, especially among Dutch-speakers.

We finally completed the moderated mediation model tested in Study 1 by including judgment about the unfairness of the repression of collaboration. Results showed that the mediating effect of this variable was stronger among mildly and strongly identified participants. Results also confirmed the moderating effect of Linguistic identification on the mediating effect of Immorality of collaboration, as well as on that of Unfairness of collaboration.

## General discussion

Since the end of WWII, diverging representations in the North and in the South of Belgium have heavily weighted on relations between the two main linguistic communities (Gotovitch & Kesteloot, 2002). Indeed, these representations continue to reappear in political debates surrounding the Belgian linguistic conflict and one can assume they will continue to condition intergroup relations in Belgium. The aim of this article was to investigate if and how some dimensions of Belgians’ attitudes towards collaboration with Germany during WWII influence standpoints in current political debates. More precisely, the goal was to compare these effects on French- and Dutch- speakers’ position-taking towards the amnesty for former collaborators issue.

Across two independent studies carried out in 2012 and in 2015, we have shown that collaboration was morally condemned, and attitudes towards amnesty were predominantly negative, in both groups. However, as we anticipated, Dutch-speaking participants were relatively more in favour of amnesty than French-speaking participants. This is understandable because they are more concerned by it due to the association, in collective memories, of their ingroup with WWII collaboration. In accordance with this suggestion, we observed differences in moral judgments about collaboration and about the repression of collaborators between the two groups: Dutch-speakers judged collaboration as less immoral and judged the repression of collaboration as more unfair than did French-speakers. However, judgments about the inappropriate management of popular vindictiveness by the State did not vary between groups.

Results of these two studies strongly suggest that contemporary political standpoints in the debate over amnesty for WWII in Belgium are, at least partly, based on diverging attitudes towards collaboration itself, and about the way it was repressed after WWII. Indeed, we found in two studies that the effect of group belonging on support for amnesty was mediated by judgments of immorality of collaboration, and study 2 showed that judgment of unfairness of repression played the opposite mediating role. However, this mediation was moderated by Linguistic identification. Dutch-speakers who identified with their linguistic group tended to judge collaboration as less immoral than French-speakers who also identified with their linguistic group, and therefore expressed more support for the amnesty of former collaborators. Moreover, mildly or highly identified Dutch-speaking participants judged repression as more unfair than did mildly or highly identified French-speakers and therefore expressed more support for amnesty. Conversely, there was no difference between French- and Dutch-speakers who weakly identified with their linguistic group.

Historians have shown that, while collaboration was associated in Wallonia with a fringe of the population characterized as “profiteers” and “thugs” (Gotovich & Kesteloot, 2002), in Flanders, it was associated with the image of an idealistic Flemish nationalist who committed a mistake by collaborating (Balace, 2002). This widespread interpretation could account for the fact that collaboration was judged as less immoral among Dutch-speaking than among French-speaking participants in our studies. Indeed, as our results revealed, this judgment plays an important role in explaining divergent attitudes towards the granting of amnesty to former collaborators. Furthermore, the repression of collaborators after the war was an important topic in post-war Flemish nationalist discourses (Gotovich & Kesteloot, 2002), in which it was often portrayed as more severe and less legitimate than in Wallonia. Our results also confirmed the influence of judgments of this repression as unfair on current standpoints towards the amnesty issue. Finally, judgments about the inadequacy of the role played by the State when presumed collaborators were brutalized or humiliated proved to play a role in legitimating claims for amnesty, especially among Dutch-speakers, but this factor did not account for differences in support for amnesty between the two groups.

Heenen-Wolff et al. (2012) consider that collaboration created an important trauma in the Flemish identity. Because holding a negative image of one’s group is unbearable (Branscombe & Doosje, 2004), demanding amnesty for former collaborators could be viewed as a means to restoring the group’s damaged identity. In contrast, granting amnesty can be perceived as threatening for the French-speaking identity, which was built on the ideal of resistance (Balace, 2002). Furthermore, pinpointing the collaborationist past of Flanders allows the preservation of a positive French-speaking identity in contrast with this shameful past of Flanders (Klein et al., 2010).

We also showed that, among Dutch-speakers, national identification had the opposite effect of linguistic identification. Indeed, the more they identified as Belgians, the more they morally condemned collaboration, the less they judged repression as unfair, the less they condemned the State, and the less they supported amnesty. This was not the case among French-speakers: identification with the linguistic group and with Belgium had similar effects on all these variables. These two identifications appear as compatible for French-speakers. Therefore, it is worth noting that national identification reduced differences between French- and Dutch-speaking respondents: high national identifiers in both groups regarded collaboration negatively, did not consider repression as unfair, did not condemn the State, and opposed amnesty. Two interpretations could account for this effect. The first is rooted in history. Collaboration was viewed as infringing the national rules and interests, whereas it was presented by some Flemish nationalists, during and after the war, as compatible with Flanders’ interests (Balace, 2002). Participants who identified with Belgium thus probably also adhere with the national framing of the collaboration issue, and therefore tend to condemn it. The second interpretation is rooted in social psychology. According to the Common ingroup identity model (Gaertner & Davidio, 2000), individuals who identify strongly with a superordinate group tend to share representations and perceive themselves as more similar to outgroup members, whereas individuals who identify primarily to subordinate groups tend to perceive themselves as more different, and also hold divergent representations. As a superordinate ingroup, identification with Belgium indeed plays such a conciliatory role, whereas identification with the linguistic subgroups has the opposite effect. These historical and social psychological explanations are obviously not mutually exclusive. It is worth noting that, among French-speakers, the two levels of identification were positively correlated: the more they felt French-speakers, the more they felt Belgian. In contrast, we obtained the opposite trend among Dutch-speakers: the more they felt Flemish, the less they felt Belgian. Moreover, political orientation had a polarizing effect on Dutch-speaking respondents: the more right-wing they were, the more they identified with the Flemish community, and the less they felt Belgian. It is noteworthy that the two levels of identification (Belgian and Flemish) crossed precisely at the centre of the political spectrum: Belgian and Flemish identities were seen as compatible among left-wingers, whereas right-wingers viewed them as incompatible.

Political orientation also had a strong impact on judgments of collaboration and standpoints about amnesty: the further right the respondents stood, the less they judged collaboration negatively. Moreover, in both studies, the more Dutch-speakers positioned themselves on the right side of the political spectrum, the more they supported amnesty. Among French-speakers, rejection of amnesty was consensual across the political spectrum, with the only exception of a small number of far-right voters.

To sum up, these two studies suggest that there is a conflict of interpretation about this dark side of Belgium’s history between French- and Dutch-speakers. French-speakers are more uncompromising towards collaboration and amnesty, while Dutch-speakers are less judgmental of collaborators during WWII and are relatively more favourable to amnesty. As illustrated by our results, these differences are polarised by participants’ identification with their linguistic group, and reduced by a superordinate identification with Belgium. This might be a result of how each community has represented its past (collective memories) and, how independently of these differences, the State has represented it.

Despite the fact that our data were collected from relatively large samples of respondents and that all age groups and political orientations were represented, these samples were not representative. Indeed, these were not random samples, and they comprised more French-speaking (60.5% in Study 1, and 61.5% in Study 2) than Dutch-speaking respondents. In addition, the first study (and to a lesser degree the second study) comprised more left-wing voters than actual election results in Flanders, where the majority usually votes for right-wing parties. The second study comprised more right-wing voters than actual election results in Wallonia, which is predominantly left-wing. However, these differences in samples were taken into account as we controlled for the effects of age, gender, and political orientations in all analyses. In addition, the two independent surveys yielded similar results, even though they were separated by a three-year interval, which testifies for their robustness. Nevertheless, these results should not be generalized to the whole populations, and should be interpreted with caution. In addition, other variables, that were not taken into account in our studies, could account for the Belgian standpoints towards the amnesty issue. Future research is needed to identify them. Finally, the cross-sectional nature of the two studies prevents us for ascertaining the directions of causality we have proposed, based on a social psychological interpretation of Belgian memories and attitudes towards WWII collaboration.

To conclude, the differences we observed in Dutch- and French-speakers’ position-taking about collaboration are probably both rooted in different war experiences and in different post-war discourses. These results provide evidence that social representations of history, and the associated judgments and attitudes, are worth taking into account for understanding current intergroup tensions. In Belgium, differences in representations of WWII collaboration contribute to current misunderstandings and fuel the intergroup conflict. A better knowledge of the history of WWII, as well as a better understanding of the other group’s interpretation and representation of this history, are necessary conditions for improving these intergroup relations, and thus for envisioning a common future.
